# The Magnetic Compass of Birds: The Role of Cryptochrome

**DOI:** 10.3389/fphys.2021.667000

**Published:** 2021-05-19

**Authors:** Roswitha Wiltschko, Christine Nießner, Wolfgang Wiltschko

**Affiliations:** FB Biowissenschaften, Goethe-Universität Frankfurt, Frankfurt am Main, Germany

**Keywords:** compass orientation, inclination compass, magnetoreception, radical pair model, cryptochrome, FAD, photocycle, UV/V cones

## Abstract

The geomagnetic field provides directional information for birds. The avian magnetic compass is an inclination compass that uses not the polarity of the magnetic field but the axial course of the field lines and their inclination in space. It works in a flexible functional window, and it requires short-wavelength light. These characteristics result from the underlying sensory mechanism based on radical pair processes in the eyes, with cryptochrome suggested as the receptor molecule. The chromophore of cryptochrome, flavin adenine dinucleotide (FAD), undergoes a photocycle, where radical pairs are formed during photo-reduction as well as during re-oxidation; behavioral data indicate that the latter is crucial for detecting magnetic directions. Five types of cryptochromes are found in the retina of birds: cryptochrome 1a (Cry1a), cryptochrome 1b, cryptochrome 2, cryptochrome 4a, and cryptochrome 4b. Because of its location in the outer segments of the ultraviolet cones with their clear oil droplets, Cry1a appears to be the most likely receptor molecule for magnetic compass information.

## The Geomagnetic Field

The geomagnetic field surrounding the Earth is generated mainly by the Earth itself. Its two poles lie close to the rotational (geographic) poles but are not identical with them. The magnetic field lines emerge from the pole in the South (which is a physical North pole), where they go straight upward, then run around the Earth and reenter its surface at the pole in the North. Near the geographic equator, they form the “magnetic equator,” being horizontal, parallel to the Earth’s surface. The angle between the field lines and the horizontal, called “inclination,” or “dip,” is defined as positive when it is downward as in the northern hemisphere and negative when it is upward; it is 0° at the magnetic equator and -90°, respectively, +90°, at the poles. Magnetic intensity (field strength) is low compared to technically applied fields; it is largest at the magnetic poles, where it is about 60 μT (microTesla) and decreases toward the magnetic equator, with a local low of 23 μT at the South American east coast. At Frankfurt am Main, Germany (50°N, 9°E), for example, the inclination today is about 67° and the total intensity about 47 μT (for more information, see Winklhofer, 2009; Finlay et al., 2010).

We humans need instruments like a technical compass for indicating directions and a magnetometer for measuring intensity. Many animals from a wide variety of groups, however, are directly sensitive to the geomagnetic field and can use its information for navigation. Birds are by far the best-studied group, and the respective findings indicate that they have two sensors for different qualities of the geomagnetic field: one based on chemical processes to obtain directional information from the course of the field lines to use as a compass and another one based on magnetite (a special form of Fe_3_O_4_) to measure magnetic intensity as a component of the navigational mechanisms indicating the location (for an overview, see, e.g., R. Wiltschko and Wiltschko, 2019). Here, we will focus on the avian magnetic compass, its functional properties and what is presently known about the detection of magnetic directional information and the putative role of the cryptochrome.

## The Avian Magnetic Compass

The ability of birds to use compass information from the magnetic field was first described in the 1960s in migratory birds. During the migratory seasons in autumn and in spring, these birds have a strong urge to move into their innate migratory direction. They show this directional preference also in suitable test cages, and when the North direction of the magnetic field around the cage is shifted by a coil system, the bird changes its heading accordingly, maintaining the same magnetic bearing. This was first demonstrated in European Robins, *Erithacus rubecula* (Turdidae), a small songbird (W. Wiltschko, 1968), but has meanwhile been shown in more than 20 other bird species from various avian lineages that are not closely related (for a list, see W. Wiltschko and Wiltschko, 2007). Among them are also a number of non-migrants, like, e.g., Homing Pigeons, *Columba livia domestica*, where it was demonstrated in homing experiments (Keeton, 1971; Walcott and Green, 1974) and by conditioning (Wilzeck et al., 2010). It was also demonstrated by directional training in Domestic Chickens, *Gallus gallus domesticus* (Phasanidae; Freire et al., 2005), and Zebra Finches, *Taeniopygia guttata*, (Estrildidae; Voss et al., 2007; Pinzon-Rodriguez and Muheim, 2017). Using the geomagnetic field as a compass thus seems to be a general feature of birds that employ this compass for orientation within their home range as well as during migration (for review, see, e.g., R. Wiltschko and Wiltschko, 2014, 2019).

The functional mode of the avian magnetic compass was also analyzed in behavioral experiments with migrating birds by exposing the test birds to different magnetic conditions and observing their responses. Three characteristics became evident:

(1) The magnetic compass of birds does not sense the polarity of the magnetic field but the axial course of the field lines. It is a so-called *inclination compass* that decides between the directions of this axis by the inclination of the field lines. For birds, the magnetic compass does not indicate magnetic North and South, as our technical compass does, but instead “poleward,” the direction where the field lines are tilted downward and “equatorward,” where they are pointing upward (W. Wiltschko and Wiltschko, 1972). This type of magnetic compass was found in all bird species tested for it (see R. Wiltschko and Wiltschko, 2014).
(2) The avian magnetic compass works only in a functional window around the local geomagnetic intensity, with fields more than about 20 to 25% weaker and stronger than the local field leading to disorientation. In particular, the disorientation in stronger fields was unexpected and seems unusual for stimuli. However, the functional window is not fixed but rather proved to be flexible: after staying in a field with higher or lower intensity, birds were able to orient at the respective intensity. European Robins caught and kept at 47 μT could adjust to fields as low as 4 μT and as high as 150 μT without losing their ability to oriented in the local field of 47 μT. (W. Wiltschko, 1978; Winklhofer et al., 2013) Yet they could not orient in an intermediate field that they had not experienced before – it appears that staying in a field outside the original functional window creates a new functional window at the respective intensity (W. Wiltschko, 1978; W. Wiltschko et al., 2006; Winklhofer et al., 2013). A similar response to magnetic intensity was found in Domestic Chickens (W. Wiltschko et al., 2007).
(3) The magnetic compass of birds is light-dependent (W. Wiltschko and Wiltschko, 1981). It works in bright sunshine under the full spectrum light (see, e.g., R. Wiltschko et al., 1981), but further studies showed that light from the short-wavelength end of the spectrum is required and that the light intensities necessary are very low. Birds were oriented under narrow-band light with a peak wavelength of 373 nm ultraviolet (UV), 424 nm blue, 501 nm turquoise, and 565 nm green light; under 568 nm and 585 nm yellow light and 617 nm, 635 nm, and 645 nm red light they were disoriented (W. Wiltschko et al., 1993; W. Wiltschko and Wiltschko, 1995, 1999; Rappl et al., 2000; Muheim et al., 2002; Pinzon-Rodriguez and Muheim, 2017). These studies were done under rather low intensities (8 × 10^16^ quanta s^–1^ m^–2^, see, e.g., R. Wiltschko et al., 2010).

## The “Radical Pair” Model

These characteristics of the avian magnetic compass imply an unusual sensory mechanism, and they caused Ritz et al. (2000) to propose the “Radical Pair”-model based on spin-chemical processes in photo-pigments that interact with the geomagnetic field. Absorption of photons leads to the formation of radical pairs, which can occur in two states, namely *singlet* with antiparallel spin and *triplet* with parallel spin, with the ratio singlet/triplet depending on the directional relation of the radical pair to the ambient magnetic field. Since the singlet and triplet products are different, this ratio could indicate magnetic directions. Ritz et al. (2000) proposed the eyes as a site of magnetoreception because light is available and because of their round shape, the receptor cells are arranged in all spatial directions. This would result in a specific activation pattern across the retina that is centrally symmetric to the direction of the magnetic vector and thus can indicate the axis of the field lines. As photo-pigment mediating magnetic directions, cryptochrome was suggested because it is the only photo-pigment known in animals that forms radical pairs.

This model can explain the specific characteristics of the avian magnetic compass: Since the relationship of the radical pair to the field lines and thus the singlet/triplet ratio ignores the polarity of the field, the consequence is an inclination compass as found in birds. The fact that the activation pattern would change with intensity explains the flexible functional window; birds experiencing a sudden intensity change are faced with a novel activation pattern. This may at first be confusing; however, because the pattern retains its central symmetry to the magnetic vector, birds can eventually learn to interpret it. The dependency of magnetic compass orientation on short-wavelength light is largely in agreement with the absorbance spectrum of cryptochrome (see, e.g., Müller and Ahmad, 2011), which was found in the avian eyes (Nießner et al., 2011, 2013).

**FIGURE 1:**
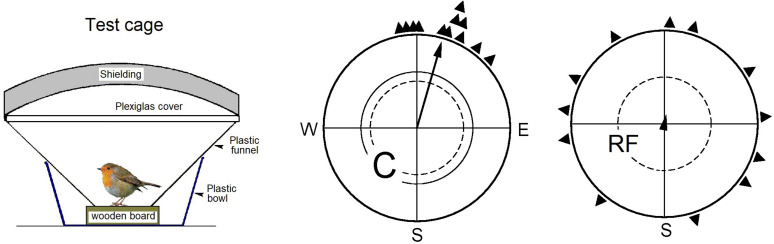
Experiments with European Robins to demonstrate the disorienting effect of radiofrequency fields, indicating the involvement of radical pair processes (see Henbest et al., 2004). Left: Bird in a cage for testing migratory birds for orientation; the funnel is lined with coated paper where the bird leaves marks as it moves. – Center and right: Results of testing the same 12 birds: center, as control (C) in the local static field only; right, with a radiofrequency field of 7.0 MHz field added vertically, i.e., in an angle of 24° to the field lines (RF). The triangles at the peripheries of the circles mark the mean headings of the individual birds based on three recordings each; the arrows represent the grand mean vectors drawn proportional to the radius of the circle, and the two inner circles mark the 5% (dotted) and the 1% significance border of the Rayleigh test. (Data from Ritz et al., 2004).

Experimental evidence meanwhile supports the Radical Pair-Model. In an electro-retinographic study, differences in responses to varying magnetic directions were recorded in the retina under blue, but not under red light (Astakhova et al., 2020). Radio-frequency fields in the MHz (MegaHertz)-range are a diagnostic test for the involvement of radical pair processes (see, e.g., Ritz, 2001; Henbest et al., 2004); such radio-frequency fields indeed cause disorientation in birds (Figure 1; e.g., Ritz et al., 2004, 2009; Thalau et al., 2005; Kavokin et al., 2014; Bojarinova et al., 2020). In a recent study, oscillating magnetic fields were applied directly to the eyes by a small battery-operated coil placed on the head of the bird; here, the birds remained oriented (Bojarinova et al., 2020). However, it is not clear whether their orientation was still based on their inclination compass or whether it involved a so-called “fixed direction response,” as they are frequently observed in situations where the normal inclination compass is disrupted (see R. Wiltschko et al., 2010 for details and discussion).

## Cryptochrome Forming Radical Pairs

**FIGURE 2:**
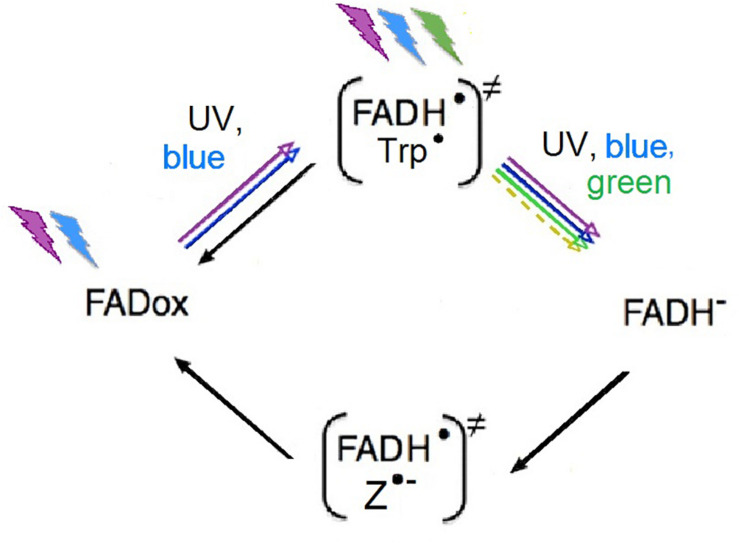
Redox-cycle of FAD, the chromophore of cryptochrome. The radical pairs are given in parentheses; colored arrows indicate photoreduction by the respective wavelengths (see text); black arrows mark light-independent reactions of re-oxidation (after Müller and Ahmad, 2011, modified). “*Z*” in the radical pair generated during re-oxidation stands for a radical whose nature is not yet clear (see text).

Cryptochromes are proteins with flavin adenine dinucleotide (FAD) as the chromophore (see, e.g., Sancar, 2003; Chaves et al., 2011). FAD undergoes a redox cycle that is best studied in plants; the oxidized form, FADox, is the dark resting form. Photon absorption of wavelengths from UV to about 500 nm blue photo-reduces FADox to the semiquinone FADH^∙^, forming a first radical pair with a tryptophan radical (Trp^∙^). FADH^∙^ can be re-oxidized independently of light, or, in a next step, it can be further photo reduced by light of wavelength from UV to green to FADH-, the fully reduced form. FADH- is then re-oxidized independently of light to FADox. During this step, a second radical pair is formed (Figure 2), possibly with O_2_^∙^ (see, e.g., Ritz et al., 2009; Müller and Ahmad, 2011), but the true partner of FADH^∙^ and details of this process are still under debate (see, e.g., Hogben et al., 2009; Lee et al., 2014; Worster et al., 2016; Kattnig, 2017; Nielsen et al., 2017; Player and Hore, 2019; Babcock and Kattnig, 2020; Procopio and Ritz, 2020).

In view of this, the question arises which of the radical pairs would mediate the direction of the magnetic field. In plants, when cryptochromes are indicating the presence and amount of light for controlling biological processes like, e.g., hypocotyl growth or flowering, the radical pair formed during photo-reduction, FADH^∙^/Trp^∙^ is considered to be the crucial one. Green light, which reduces the amount of FADH^∙^ by further photo-reducing FADH^∙^ to the fully reduced state FADH-, was found to act antagonistically to blue light *in vitro* as well as in living plants (e.g., Banerjee et al., 2007; Bouly et al., 2007). However, in the case of the avian magnetoreception, the situation is different. Many birds—European Robins, Australian Silvereyes, *Zosterops l. lateralis*, Garden Warblers, *Sylvia borin*, and Zebra Finches—are oriented under narrow-band lights with peak wavelengths at blue as well as green light (W. Wiltschko et al., 1993, W. Wiltschko and Wiltschko, 1995; Rappl et al., 2000; Pinzon-Rodriguez and Muheim, 2017). Since green light cannot photo-reduce FADox, this speaks against the involvement of the radical pair formed during photo-reduction being the one mediating magnetoreception but points to the other radical pair (see Figure 2).

Further behavioral experiments, however, showed that the orientation under green light is a transient phenomenon—it was possible only for less than 1 h. After this time, the birds were disoriented, whereas they continued to be oriented under the blue and turquoise light, that is, under wavelengths that can be absorbed by oxidized flavin. Also, orientation under green light was possible only when the birds had been before under “white” light, which included wavelengths that could photo-reduce oxidized cryptochrome—after staying in total darkness for 1 h, the birds were disoriented under green light, but they showed oriented headings under blue and turquoise light where the entire FAD-cycle can run (R. Wiltschko et al., 2014). This suggests that avian orientation under green light is possible only as long as there is a certain supply of FADH^∙^ available for further photo-reduction to the fully reduced FADH- by green light and again implies that the radical pair formed during light-independent re-oxidation is the one that mediates magnetic directions in birds.

**FIGURE 3:**
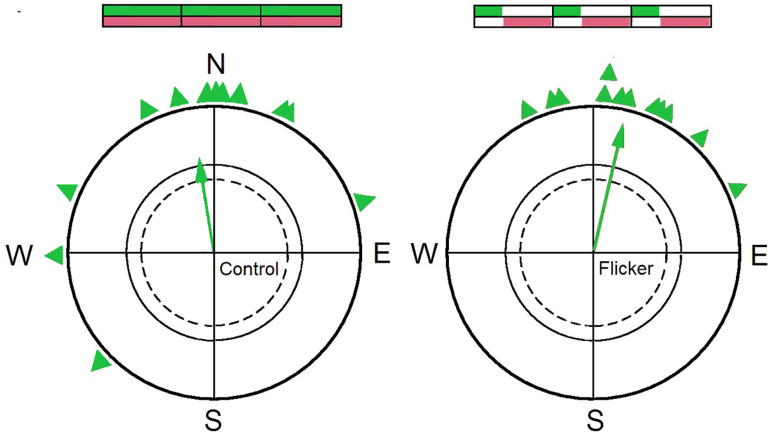
Orientation of a group of 12 birds during spring migration. Left: under control conditions with continuous light and the magnetic field permanently present, the birds are significantly oriented in their northerly migratory direction. Right: under *flicker* conditions, when the magnetic field and light was present alternatingly, the same birds are likewise oriented in their migratory direction (for details, see text). The schemes above the circles symbolize the distribution of light (above, green) and magnetic field (below; brown). Symbols as in Figure 1 (Data from R. Wiltschko et al., 2016).

To test this hypothesis, birds were exposed to flickering light and a pulsed magnetic field in the following manner: With a frequency of 1 Hz, light was available for 300 ms with the magnetic field compensated; during the remaining time, there was total darkness, but, except for security intervals of 10 ms at the beginning and the end, the geomagnetic field was present. In this situation, the birds showed oriented behavior (Figure 3; R. Wiltschko et al., 2016). This clearly shows that the actual process of sensing magnetic directions can occur in darkness—it identifies the radical pair formed during re-oxidation as the crucial one for sensing directions. Light, however, is necessary for photo-reduction to provide the fully reduced form FADH- that is re-oxidized for the formation of this radical pair.

This is different from what is known about cryptochrome when it controls light-dependent processes in plants; here, the radical pair FADH^∙^/Trp^∙^ is important because FADH^∙^ is formed, which was found to be the crucial form to indicate light. In the case of avian magnetoreception, however, radical pairs are to indicate the direction of the (geo)magnetic field. This is a different task, and it seems conceivable that evolution developed a modified mechanism that is better suited for this function.

In the future, it may help our understanding of cryptochromes and their functions if more attention was given to the re-oxidation process. A recent study (Pooam et al., 2019) suggests that magnetic effects in plants can also be mediated by processes during re-oxidation in the dark; yet here, not the direction, but the intensity of the magnetic field was effective.

## Cryptochromes in the Avian Eyes

In the eyes of birds, five types of cryptochromes – cryptochrome 1a (Cry1a), cryptochrome 1b (Cry1b), cryptochrome 2 (Cry2), Cryptochrome 4a (Cry4a), and Cryptochrome 4b (Cry4b) – have been found (see also R. Wiltschko and Wiltschko, 2019).

**FIGURE 4:**
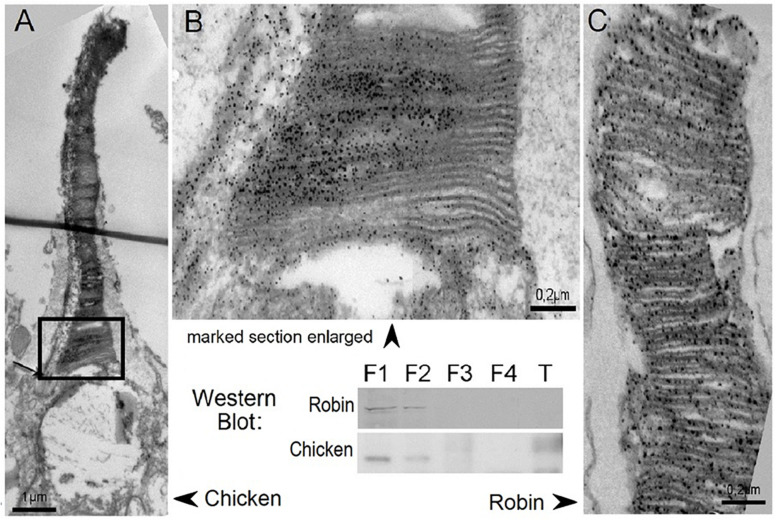
Cry1a immuno-labeled in the outer segments of the UV/V cones of chickens and robins. Electron-microscopic images of the outer segments of the UV/V-cones, with labeled Cry1a visible as dark dots along the disk membranes. **(A)** Entire outer segment of a chicken V-cone. **(B)** Higher magnifycation of the lower part of this outer segment. **(C)** Part of the outer segment of the UV-cone of a robin. -Western blots of retinae showing Cry1a in the cytosol (F1) and membrane fraction (F2); F3, nuclear fraction; F4, cytoskeletal fraction; and T, tongue tissue as control (from Nießner et al., 2011).

Cryptochrome 1 was first described in birds by Haque et al. (2002) based on mRNA expression in the photoreceptor layer and the ganglion cell layer of chickens (see also Fu et al., 2002; Mouritsen et al., 2004). In Robins, Möller et al. (2004) identified two splice products of the *Cry1* gene, Cry1a and Cry1b; they differ in their *C*-termini, which suggests different functions. In an immuno-histochemical study, Nießner et al. (2011) located Cry1a in the outer segments of the ultraviolet/violet (UVS/VS, SW1) cones in the retinae of Chickens and European Robins where immuno-marking for electron microscopy shows it positioned along the disks, where also the UV-opsin is located (Figure 4). Bolte et al. (2021) described Cry1a in the outer segments of the UV/V cones also in pigeons, Blackcaps, *Sylvia atricapilla*, and Zebra Finches, so Cry1a in these locations appears to be common in all birds. Cell fractionation revealed Cry1a in the membrane fraction, suggesting that Cry1a is anchored along membranes (Nießner et al., 2011). In birds, photoreceptor cells contain oil droplets that absorb different amounts of short wavelengths in order to shift the maximum absorbance of the color receptors further apart for better color vision. Only the UVS/VS cones have clear oil droplets that allow all wavelengths of light to pass (see Hart, 2001). This type of photoreceptor is present all across the retina, i.e., in different directional relationships to the magnetic vector; hence they can give rise to the activation pattern suggested by Ritz et al.(2000).

**FIGURE 5:**
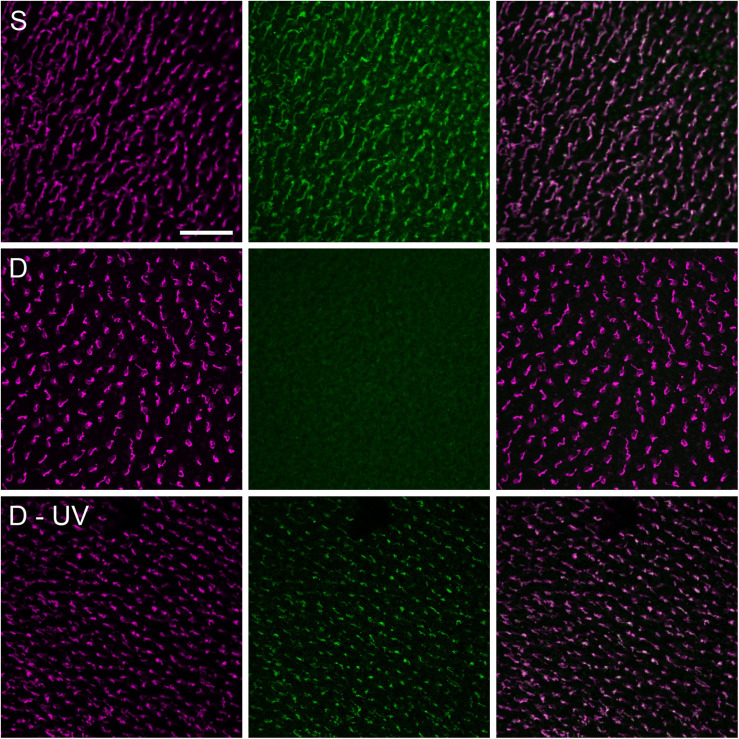
Double immuno-fluorescence labeling of chicken retinae for violet (SW1) opsin marking the violet cones (left column, magenta fluorescence) and that of Cry1a with a specific antiserum against the *C*-terminus (center column, green fluorescence), and merging of the two labels (right column). The images in each row show the labels in the same patch of the retina; with the merge indicating that Cry1a occurs only together with the violet opsin in the SW1 (violet) cones. Treatment of the birds: Upper row, *S*: pre-treatment in daylight: 30 min in sunlight: Cry1a is labeled. Center row, *D*: pre-treatment in daylight, 30 min in total darkness: no Cry1a label is visible, indicating that the antiserum only recognizes activated Cry1a. Bottom row, D-UV: 30 min pre-treatment in darkness, 5 min in 373 nm UV light: Cry 1a is labeled, showing that Cry1a has been activated by light. The scale bar represents 50 μm applies to all panels (data from Nießner et al., 2013).

Based on studies with purified Cry1, Kutta et al. (2017) questioned whether vertebrate Cry1 indeed had FAD bound and hence could be photo-reduced (see also Hochstoeger et al., 2020), although Liedvogel et al. (2007) reported that avian Cry 1a contained FAD and could absorb blue light. An *in vivo* study also showed that at least avian Cry1a is directly activated by light (see Figure 5). It is activated by all wavelengths that are absorbed by flavin: An immuno-chemical study showed activated Cry1a under all light conditions where birds were oriented (Nießner et al., 2013). Yet Bolte et al. (2021) failed to reproduce this finding; the reasons are unknown.

In contrast to Cry1a, Cry1b was located by immuno-histochemistry in the cytosol of ganglion cells, displaced ganglion cells, and also in the inner segments of the photoreceptors, free as well as bound to membranes (Mouritsen et al., 2004; Bolte et al., 2016; Nießner et al., 2016). In night-migrating birds, its expression varies with the season; it was much stronger during the migratory season when the birds were active during the night (Fusani et al., 2014; Nießner et al., 2016). It has been speculated that Cry1b possibly plays a role in magnetoreception (Bolte et al., 2016), but, because the use of a magnetic compass appears to be a general sensory ability of birds, also shared by non-migrants (see above), the seasonal changes suggest another role—one that possibly involves the shift from diurnal activity to nocturnal activity (*Zugunruhe*) by night migrants.

Bailey et al. (2002) and Fu et al. (2002) described Cry2; identifying it by its mRNA in chickens and Japanese Quails, *Coturnix japonica* (Phasanidae). It was found in a number of organs, among them the pineal gland and, in the eyes, in the photoreceptors, and in ganglion cells. This cryptochrome includes a sequence that suggests a location in the cell nucleus (Möller et al., 2004; Mouritsen et al., 2004), indicating a possible role as clock protein (see, e.g., Sancar, 2003 for discussion of this role of cryptochromes).

Cryptochrome 4 was described in the retina of chickens first by Watari et al. (2012) using mRNA-expression and immuno-histochemistry (see also Pinzon-Rodriguez et al., 2018). Günther et al. (2018) could identify Cry4 in the outer segments of the longwave-sensitive (LWS) single cones and of the double cones of European Robins. Recently, an isoform of Cry4, Cry4b, was identified by analyzing the DNA sequence. This splice product appears to be widespread; by genomic BLAST database search, it was also indicated in more than 25 avian species of various orders (Einwich et al., 2020). Another study localized Cry4 also in the outer plexiform layer, where it seemed associated with the synapses between photoreceptors and horizontal cells (Hochstoeger et al., 2020).

A possible role of Cry4 in magnetoreception has been frequently discussed (e.g., Günther et al., 2018; Pinzon-Rodriguez et al., 2018; Hochstoeger et al., 2020). Especially its location within the double cones prompted speculations on such a role of Cry4 because of the possibility that the input of two adjacent receptors with the magneto-receptive molecules oriented in different directions could be compared; it was proposed that this might help to overcome problems with different light intensities and polarization (Worster et al., 2017; Günther et al., 2018). However, the principal cone is associated with an oil droplet that acts as a cut-off filter absorbing short wavelengths (Hart, 2001) and thus most of the wavelengths required for cryptochrome photo-reduction. This, together with the gap junctions between the two cones, would interfere with a comparison, making such a role of Cry4 in the double cones rather unlikely. Hochstoeger et al. (2020) speculated about a possible role of Cry4 in the outer plexiform layer. The Cry4 in the LWS single cones seems even less suitable for magnetoreception because the red oil droplets in these cones transmit only long wavelengths that cannot photo-reduce cryptochrome. Moreover, a study by Mitsui et al. (2015) implies that Cry 4 requires a very long time for re-oxidation, which would not be favorable for receptive processes.

Altogether, if cryptochrome is indeed the receptor molecule in birds, the presently available evidence indicates Cry1a as the most likely receptor for sensing directions. The observation that its gene expression shows a diurnal rhythm (Pinzon-Rodriguez et al., 2018) does not speak against such a role, because it only indicates a rhythmic production in the inner segment of the cones, but it does not say anything about its general availability in the sensory active outer segment and its use. It appears to be a parallel case to vision, where the production of the visual pigment opsin also shows a diurnal pattern (e.g., Pierce et al., 1993; von Schantz et al., 1999), but animals can see the entire day.

## Outlook

More than 50 years after the discovery of the avian magnetic compass, we finally have a concrete idea about the primary mechanism leading to the detection of directions by the magnetic field in birds: a radical pair process, with cryptochrome playing a crucial role in this process. Yet there are still several conflicting findings that have to be resolved, and a number of open questions that have to be answered.

One of the most important questions concerns the mechanisms by which the information obtained by the radical pair is transformed into a biological signal. Cryptochrome is found in the outer segments of cones, i.e., in a cell type that is also important for color vision. Is the visual information from the opsin and information on magnetic directions from cryptochrome transmitted separately or together? One might expect the latter since the cone has only one known way of transmitting information. This would mean, however, the two types of information have somehow to be separated, either already in the eyes or later in higher centers in the brain. There are speculations about possibilities (see, e.g., Bischof et al., 2011), but it will require many more considerations and experiments until we hopefully reach a complete understanding of how birds perceive the direction of the geomagnetic field.

## Author Contributions

RW and WW wrote this review together, CN checked the text and contributed the histological data. All authors discussed the article and approved the submitted version.

## Conflict of Interest

The authors declare that the research was conducted in the absence of any commercial or financial relationships that could be construed as a potential conflict of interest.
